# Intraoperative quantification of meningioma cell proliferation potential using rapid flow cytometry reveals intratumoral heterogeneity

**DOI:** 10.1002/cam4.2178

**Published:** 2019-04-16

**Authors:** Soichi Oya, Shinsuke Yoshida, Tsukasa Tsuchiya, Naoaki Fujisawa, Akitake Mukasa, Hirofumi Nakatomi, Nobuhito Saito, Toru Matsui

**Affiliations:** ^1^ Department of Neurosurgery Saitama Medical Center, Saitama Medical University Saitama Japan; ^2^ Department of Neurosurgery Graduate School of Medicine, The University of Tokyo Tokyo Japan

**Keywords:** flow cytometry, meningioma, MIB‐1, proliferative ability, recurrence

## Abstract

**Background:**

Standard sampling methods to evaluate the proliferative ability of meningioma have not been established.

**Methods:**

This prospective study was conducted to evaluate the effectiveness of intraoperative rapid flow cytometry (iFC) using raw samples for the quantitative assessment of proliferative ability in meningioma cells and to investigate intratumoral heterogeneity. Proliferation index (PI) was defined as the ratio of aneuploid cells with an abnormal number of chromosomes to the total cells.

**Results:**

From 50 patients, 118 specimens were analyzed. There was a statistically significant correlation between the postoperative MIB‐1 labeling index (LI) and PI (R = 0.59, *P* < 0.0001). A higher PI was correlated with a higher annual growth rate (AGR, cm^3^/y) (R = 0.50, *P* = 0.0002, 26 patients). AGR showed a correlation with the intratumoral distribution of PI. PI was the highest at the center or the peripheral section of the tumor in tumors with high AGR, whereas it was highest at the dural attachment in tumors with low AGR (*P* = 0.039, n = 20). Pial feeders were more frequently observed when PI was high in the center or in the peripheral section (*P* = 0.006, n = 37).

**Conclusions:**

Rapid iFC may thus become a substitute for MIB‐1 LI. Intratumoral heterogeneity of cellular proliferative potential exists in meningiomas and is related to tumor biological characteristics such as AGR and development of pial feeders. This observation underscores the importance of standardization in the sampling method to accurately estimate the risk of meningioma recurrence.

**Abbreviations**
AGR: annual growth rate
iFC: intraoperative flow cytometry
LI: labeling index
MR: magnetic resonance
PI: proliferation index
WHO: World Health Organization

## 1 INTRODUCTION

Radical resection is usually the best strategy in meningioma surgery for long‐term tumor control and maintenance of neurological functions.^1^, ^2^ However, some meningiomas are not amenable to aggressive resection due to their severe adhesion to critical structures such as cranial nerves, arteries, and veins, as well as brain invasion. In such cases, surgeons are required to properly weigh the risks of postoperative complications and the benefits of aggressive resection to maximize the benefits to the patient without having specific pathological information.

There has been recent accumulation of knowledge regarding meningiomas based on detailed radiological and pathological examinations. One discovery is that World Health Organization (WHO) Grade 1 meningiomas believed to be uniformly benign are not necessarily homogeneous in terms of postoperative behavior.^3^, ^4^ Recent studies have led to the critical finding that the biological behavior of each tumor may have an equivalent or greater impact on the recurrence rate compared to that of the extent of the resection.^3^, ^4^, ^5^ As one indicator of biological characteristics, the MIB‐1 labeling index (LI) has prognostic value for meningioma recurrence.^1^, ^3^, ^4^, ^6^, ^7^, ^8^, ^9^, ^10^, ^11^, ^12^ Tumors with an MIB‐1 LI of 3% or higher carry a significant risk of shorter recurrence‐free survival, even after gross total resection.^3^, ^4^ However, there are some operational problems regarding the utilization of MIB‐1 LI. The surgical strategy cannot be modified during surgery based on the MIB‐1 LI result because intraoperative rapid immunohistochemistry is not usually available during surgery.^13^, ^14^ Quantification of the MIB‐1 LI is not impervious to interobserver biases.^15^, ^16^ Furthermore, an important question that remains unanswered is whether the evaluation of resected specimens truly reflects the proliferative potential of residual tumors. Therefore, establishment of a standard sampling method that can describe meningioma behavior in a reproducible and predictive way is desired.^4^, ^16^, ^18^, ^19^

To solve these questions, the possible usefulness of rapid quantification of meningioma cell proliferation potential using intraoperative flow cytometry (iFC) has been explored. Using iFC, the proportion of cells containing abnormal DNA content (aneuploid cells) relative to the entire cell population can be quickly measured intraoperatively. This study showed a close relationship between the MIB‐1 LI and the result of iFC. In addition, the highly quantitative performance of iFC demonstrated gradation in the cell proliferation in meningiomas and its association with their biological characteristics such as annual growth rate (AGR) and development of pial feeders.

## 2 MATERIALS AND METHODS

### 2.1 Patient population and tumor characteristics

This study was conducted with the approval of the institutional review board (No. 1148‐II). Fifty patients who had intracranial meningiomas for which surgical resection was planned were prospectively enrolled. Patients who had recurrent meningiomas, previously radiated meningiomas, and meningiomas treated by preoperative embolization were excluded. Between January 2015 and September 2017, 58 patients with intracranial meningiomas underwent surgery at our institution. After excluding three patients with recurrent meningiomas, two with previously irradiated meningiomas, two with meningiomas with preoperative embolization, and one with intraosseous meningioma, 50 patients were enrolled and provided informed consent. Of these, 26 tumors were followed for more than 6 months before the surgery, and volumetric analysis based on serial magnetic resonance (MR) imaging was conducted to investigate the relationship between the absolute growth rate and proliferation index (PI).

The tumors were resected in a standard fashion. In each surgery, a 5‐mm specimen of the tumor was obtained and equally dichotomized. For 37 large tumors with a maximum diameter of 2.5 cm or greater, specimens were taken from three different locations, namely, the region close to the dural attachment, the center of the tumor, and the peripheral region of the tumor in contact with the brain surface. One specimen was sent to pathology for routine histological diagnosis and the other specimen was used for iFC. The presence of pial feeders was assessed during surgery by the first author (SO).

### 2.2 Intraoperative flow cytometry and histological analysis

**Figure 1:**
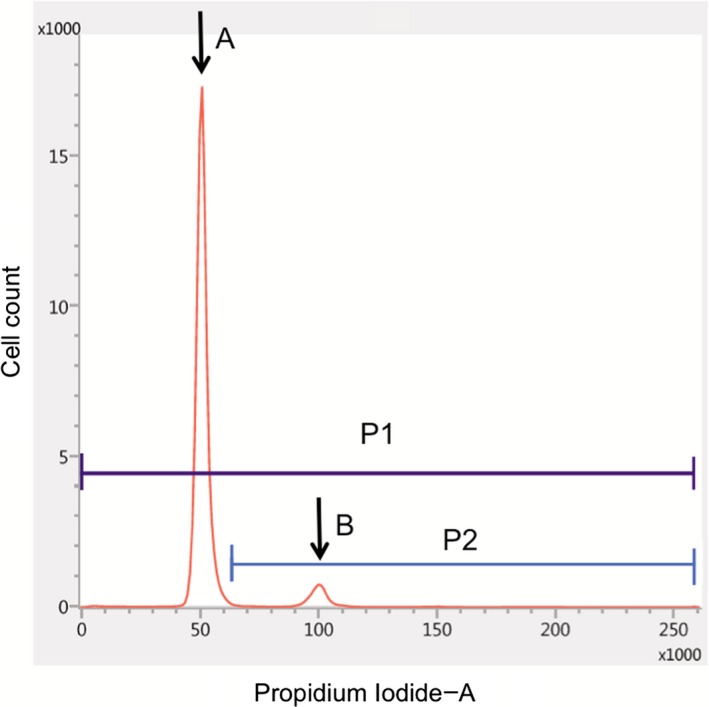
Analysis of DNA ploidy using flow cytometry. The horizontal axis is the intensity of propidium iodide fluorescence. Peak A represents the cluster of G_0_G_1_‐phase (euploid) cells, whereas Peak B denotes that of aneuploid cells with an abnormal number of chromosomes and G_2_/M‐phase cells. Proliferation index (PI) was defined as the ratio of the number of cells with greater than normal DNA content (P2) to the total number of cells (P1)

One approximately 2‐mm‐sized specimen obtained during surgery was sent to the laboratory in our institution within an hour. It was placed in a microtube and immersed in a kit solution (DNA Peak; Nihon Kohden Corporation, Tokyo, Japan). The specimen was then disrupted by repetitive pipetting for 200 seconds. The homogenized sample was transferred into another microtube and mixed with a surface‐acting agent to stain the cell nuclei at room temperature. The suspension was filtered through a 50‐µm nylon mesh and the DNA content was measured using a BD FACSverse^TM^ flow cytometer (Becton Dickinson Biosciences, Franklin Lakes, NJ) to obtain the DNA histogram (Figure 1). Each area of the histogram was interpreted following a previous study reported by Shioyama et al^20^ Namely, Peak A indicated G_0_G_1_‐phase (euploid) cells. Cells in the area to the left of Peak A were the sum of sub‐G_0_G_1_‐phase cells, apoptotic cells, and debris. Peak B represented aneuploid cells with an abnormal number of chromosomes and G_2_/M‐phase cells. Cells falling in the interval between Peaks A and B were in the S phase. PI was calculated as the ratio of the number of cells with greater than normal DNA content to that of the total number of cells to investigate the proliferative potential of each meningioma. This index corresponded to the “malignancy index” in the report by Shioyama et al^20^ The word “malignancy” was not necessarily appropriate for mostly benign meningiomas. Therefore, we renamed this index as PI in this study for convenience. The actual time required for flow cytometry was approximately 10 minutes.

Pathological diagnosis was made in the Department of Pathology at our institution based on the 2007 edition of the WHO Classification of Tumors of the Central Nervous System.^21^ The MIB‐1 LI was calculated in a blind fashion using the highest LI method in the areas of maximum density, as identified by visual analysis.^18^

### 2.3 Volumetric analysis

Volumetric analysis for preoperative tumor growth was conducted for 20 patients (40%) who underwent MR imaging at our institution or in other hospitals prior to surgery, at a minimum of two different time points with an interval of 6 months or longer. The exact procedure adopted for the volumetric analysis is reported elsewhere.^22^ Briefly, we utilized the radiological data stored in the form of DICOM (Digital Imaging and Communications in Medicine) files and measured the volume change in each tumor using ImageJ Version 1.50i (https://rsbweb.nih.gov/ij/) in a blinded manner. The AGR (cm^3^/y) was calculated by dividing the absolute volume change by the length of the interval between the first and last MR images.

### 2.4 Statistical analysis

The non‐parametric Spearman's correlation coefficient method was used to evaluate the statistical significance of the correlation between MIB‐1 LI, AGR, and PI. Fisher's exact test was used to compare the categorical variables. An unpaired Student's *t* test was used for comparing the means of continuous variables. All analyses were performed using JMP 9.0.0 (SAS Institute, Cary, NC). A *P* value of <0.05 was considered statistically significant.

## 3 RESULTS

The patient demographics and tumor characteristics are listed in Table 1. From 50 tumors in 50 patients, 118 specimens were obtained. The mean age was 64.0 (range, 35 − 85) years. Thirty‐two patients were women (64%). Skull base meningiomas comprised 44% of all tumors. Using the WHO classification, 40 tumors were classified as Grade I, nine as Grade II, and one as Grade III. The mean MIB‐1 LI was 3.5% (0.5‐20.1). The mean maximum diameter was 3.9 cm (1.5‐8.1), including 37 tumors (74%) with a diameter of 2.5 cm or larger.

**Table 1:** Patient demographics and characteristic of tumors

Factor	Value
No. of patients	50
No. of tumors	50
No. of specimens	118
Mean age (range)	64.0 (35‐85)
Sex	
Male	17
Female	32
Mean tumor diameter (cm)	3.9 (1.5‐8.1)
Tumor location	
Skull base	22
Non‐skull base	28
WHO Grade	
Grade I	40
Grade II	9
Grade III	1
Mean MIB‐1 labeling index (range)	3.5% (0.5‐20.1)
No. of tumors with a size of 2.5cm or larger	37/50 (74.0%)

### 3.1 The results of iFC correlated with the existing indices of proliferation and WHO grading

**Figure 2:**
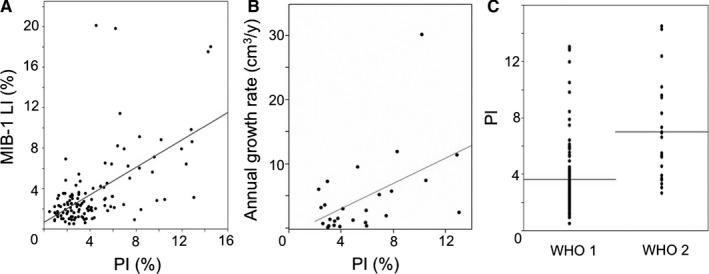
Correlation between the proliferation index (PI) and the existing indices for proliferation and recurrence; MIB‐1 labeling index (LI) (A, R = 0.59, *P* < 0.0001), annual growth rate (cm^3^/y) (B, R = 0.50, *P* = 0.0002), and World Health Organization (WHO) grades (C, average 3.62% vs 6.99%, *P* < 0.0001)

We investigated the association between the PI obtained by iFC and the representative marker for proliferation, namely, the MIB‐1 LI calculated from permanent specimens. There was a moderate correlation between the MIB‐1 LI and the PI measured intraoperatively (Figure 2A; R = 0.59, *P* < 0.0001). AGR was calculated for 26 tumors for which serial MR imaging prior to surgery had been conducted more than twice with an interval of 6 months or longer. The correlation between high PI and high AGR was statistically significant (Figure 2B; R = 0.50, *P* = 0.0002). When tumors were divided into WHO Grades I and II, the specimens from Grade II meningiomas showed significantly higher PI than those from Grade I meningiomas (Figure 2C; average, 3.62% vs 6.99%, respectively, *P* < 0.0001). These results indicate the applicability of PI determined by iFC as a marker for proliferative potential.

### 3.2 Intratumoral heterogeneity of PI was related to tumor biological characteristics such as AGR and the development of pial feeders

**Figure 3:**
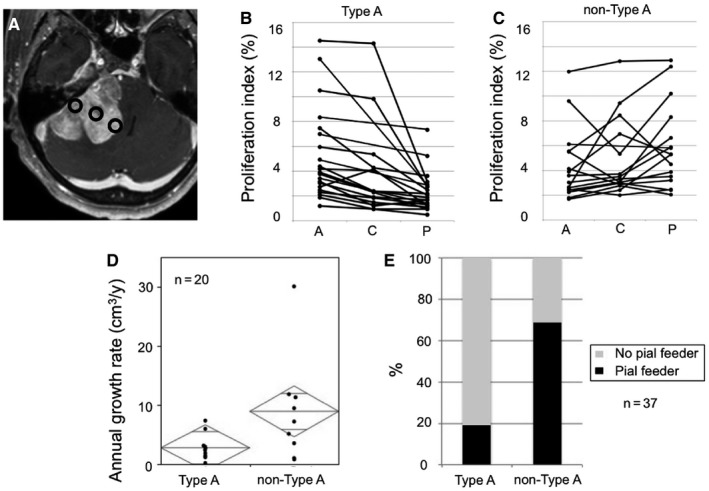
Intratumoral heterogeneity of meningiomas. A, An example of sites to obtain specimens. For tumors with a size of 2.5 cm or larger, specimens were obtained from the attachment, center, and peripheral sections (circles) of the tumor. B, Among 37 large tumors (2.5 cm or larger in maximum diameter), 21 tumors for which the proliferation index (PI) is the highest in the attachment region were classified as Type A tumors. The graph shows the PI of each Type A tumor at the attachment region (A), central section (C), and peripheral section (P). C, Sixteen tumors for which the PI was the highest at the central or peripheral sections were grouped as non‐Type A tumors. D, The annual growth rate (AGR) was significantly higher in tumors with the highest PI in the center or peripheral regions (non‐Type A tumors) than in those with the highest PI in the attachment (Type A tumors). Mid‐bars in diamonds represent the mean. Heights of diamonds indicate 95% confidence interval. E, The development of pial feeders was more frequently observed among non‐Type A tumors than in Type A tumors

For 37 large tumors (2.5 cm or larger in maximum diameter), samples were obtained from the attached, central, and peripheral section of the tumors (Figure 3A). The peripheral area was intraoperatively determined as the farthest part from the attachment based on the preoperative MR imaging. When the PI was the highest at the attachment, the tumor was classified as Type A (Figure 3B). On the other hand, 16 tumors with the highest PI in the central or the peripheral areas were grouped as non‐Type A tumors (Figure 3C). There were 20 large meningiomas for which preoperative serial MR imaging was available. AGRs of Type A tumors were significantly lower than those of non‐Type A tumors (Figure 3D; *P* = 0.039). In addition, non‐Type A tumors were more frequently associated with the presence of pial feeders compared to Type A tumors (Figure 3E; *P* = 0.006).

### 3.3 Illustrative cases

#### 3.3.1 Case 1

**Figure 4:**
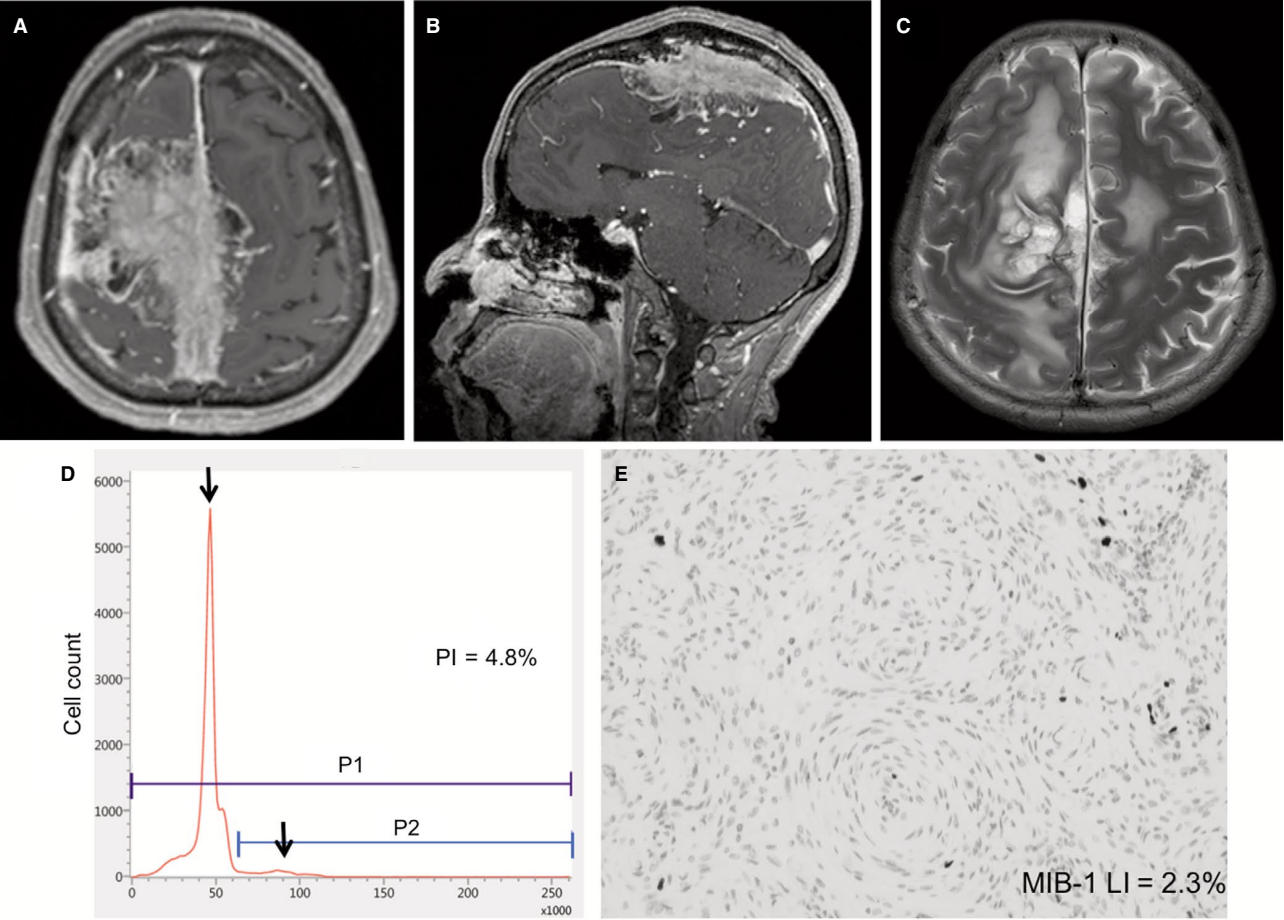
Illustrative case 1. Preoperative axial (A) and sagittal (B) T1‐weighted magnetic resonance (MR) images with gadolinium enhancement showing a right parasagittal meningioma invading the superior sagittal sinus and the parietal bone. Axial T2‐weighted MR image demonstrating significant peritumoral edema (C). The radiological characteristics were consistent with a WHO Grade II meningioma, but the proliferation index (PI) was relatively low (4.8%) by the intraoperative flow cytometry (D). The postoperative histological diagnosis was WHO Grade I meningioma with an MIB‐1 labeling index (LI) of 2.3% (E). Original magnification, **×**200. P1, the total area of cells; P2, the area of cells with greater than normal DNA content

A 66‐year‐old woman had a parasagittal meningioma found incidentally (Figure 4A). The tumor was accompanied by extensive invasion into the superior sagittal sinus and the skull (Figure 4B), with peritumoral edema (Figure 4C). Although the preoperative diagnosis was a meningioma of WHO Grade II or higher, the intraoperative frozen‐section indicated a diagnosis of benign meningioma of WHO Grade I. The intraoperative iFC revealed relatively low PI (Figure 4D, 4.8%). Due to severe adhesion, some small pieces strongly adhering to the cortical arteries and veins were therefore intentionally left. Postoperative immunohistochemistry showed low MIB‐1 LI (2.3%; Figure 4E). The patient was discharged with mild weakness in the right leg. Follow‐up MR images obtained at 3 years after the surgery showed no sign of recurrence.

#### 3.3.2 Case 2

**Figure 5:**
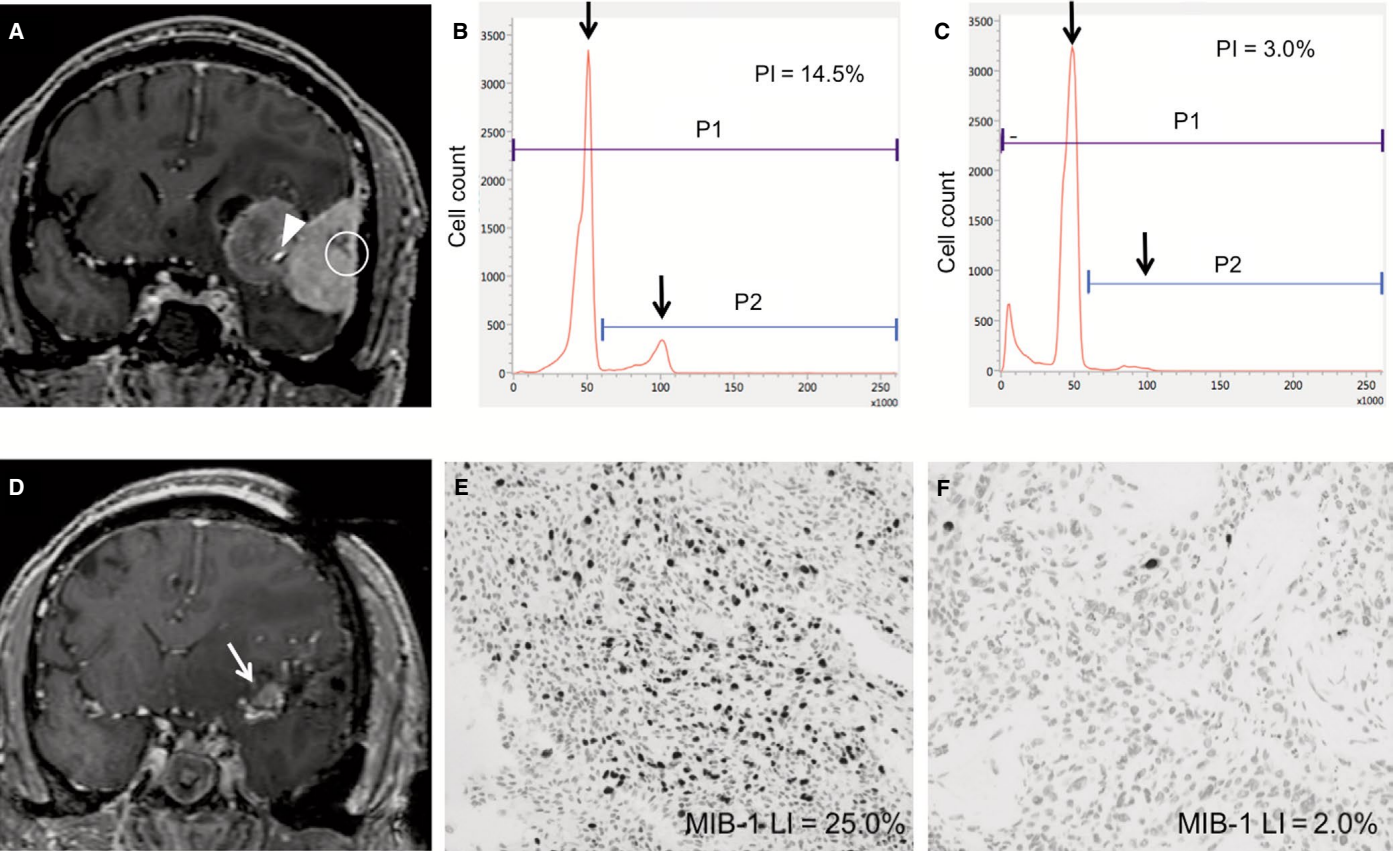
Illustrative case 2. Coronal T1‐weighted magnetic resonance image with gadolinium enhancement (A) revealing an irregular‐shaped mass along the left sphenoid wing. The specimens were obtained from the attachment (circle) and the middle cerebral artery (MCA) bifurcation (arrowhead). The proliferation index (PI) of the specimen obtained from the attachment was 14.5% (B). However, the specimen near the MCA bifurcation had a low PI of 3.0% (C). Due to severe adhesion, a small mass firmly adhering to the MCA bifurcation (arrow) was left intentionally (D, arrow). Postoperative pathological analysis revealed that the MIB‐1 labeling index (LI) was elevated to 25.0% in the attachment (E) and 2.0% in the MCA bifurcation (F). Original magnifications in C and D, **×**200. P1, the total area of cells; P2, the area of cells with greater than normal DNA content

A 56‐year‐old man suffered from generalized seizure and was diagnosed with an irregularly shaped sphenoid ridge meningioma on the left side. During surgery, iFC indicated that the PI of the specimen obtained from the dural attachment (Figure 5A, circle) was elevated to 14.5% (Figure 5B). In contrast, the PI of the specimen from the part encasing the middle cerebral artery (MCA) bifurcation (Figure 5A, arrowhead) was much lower (3.0%) than that of the attachment (Figure 5C). Because the tumor was severely adhered to the MCA bifurcation, this small residue was left to avoid major neurological deficits (Figure 5D, arrow). Postoperative histological diagnosis was atypical meningioma of WHO Grade II. The MIB‐1 LI was 25.0% at the attachment (Figure 5E) and 2.0% near the MCA bifurcation (Figure 5F). After adjuvant radiation to the attachment and residual mass, the patient was discharged with no neurological deficit. No tumor growth was observed during a 1.5‐year follow‐up.

## 4 DISCUSSION

Our data demonstrate two novel findings. First, rapid iFC can be used as a substitute for MIB‐1 LI, which may contribute to achieving safe maximal resection. Second, cellular proliferative potential shows that intratumoral heterogeneity exists in meningiomas and is related to their biological characteristics such as AGR and the development of pial feeders.

The extent of resection evaluated by the Simpson grade has been playing a pivotal role in predicting the recurrence of meningioma,^23^ which has been proven effective even in the current advanced surgical techniques. Following this doctrine, it is advocated that gross total resection is usually the best strategy in the treatment of meningiomas because it minimizes the risk of recurrence and assures long‐term preservation of neurological functions.^1^, ^2^, ^24^ However, aggressive resection is not always achievable. Moreover, the effort to maximize the resection rate may have reached a plateau due to the recent advancement in surgical devices and techniques. A small amount of residue after resection of histologically benign meningiomas can be safely controlled nowadays by stereotactic radiation. The recent reappraisal of the clinical significance of the resection rate on long‐term tumor control^1^, ^2^, ^4^, ^24^, ^25^ reflects the recent awareness that the biological characteristics of each tumor should be properly assessed to implement personalized treatment. MIB‐1 LI has been well established for assessing the proliferative ability of meningioma cells and predicting recurrence.^1^, ^4^, ^27^

Weighing the risks and benefits of aggressive resections and determining treatment strategy require the integration of multiple factors. Although preoperative MR imaging provides some clues to assess invasiveness, a discrepancy between the radiological and histological findings can occur, as shown in our illustrative case 1. MIB‐1 staining is quite helpful to recognize the proliferative ability of meningioma cells, but surgical strategy cannot be modified based on this information because it is unavailable during surgery. Therefore, surgeons must make every effort to seek the best balance between the risks of surgery and the benefits of aggressive resection during surgery based on empirical evaluation regarding tumor adhesion to the cranial nerves, encasement of large vessels, pial invasion, and other variables.

To overcome these current limitations, the present study was initiated to assess the value of iFC in the treatment of meningiomas. There have been some pilot studies regarding the effectiveness of flow cytometry in investigating the biological aggressiveness of meningiomas.^17^, ^28^, ^29^ With the aid of a newly developed kit solution, iFC has become readily available.^20^ Our results show that the PI obtained intraoperatively is significantly associated with the MIB‐1 LI calculated postoperatively. A recent study reporting the usefulness of iFC in determining WHO Grades of meningiomas also supports our results.^30^ Taking advantage of this information, surgeons may be able to refine the surgical strategy in a more individualized manner. For meningiomas with low proliferative potential, it would be safer and reasonable to avoid high‐risk dissection of tumors adherent to the cranial nerves, vessels, and brain parenchyma. In contrast, extensive resection of the affected bone and dura or aggressive resection using vascular reconstruction techniques may be required for meningiomas with high risk of early recurrence. In addition, the determination of the surgical strategy should not be based solely on the results of iFC. This hypothesis needs to be validated with a detailed evaluation of functional preservation and long‐term follow‐up for tumor recurrence, which is beyond the scope of this non‐interventional study.

In addition to its rapidness, high quantitative performance is another advantage of iFC. Several previous research studies have noted that calculation of MIB‐1 LI is not exempt from inter‐ and intra‐observer bias,^15^, ^16^, ^31^ thus necessitating more quantifiable and reproducible methods. Evaluation of proliferative ability using MIB‐1 staining is associated with some arbitrary processes, including how to choose the specific fields to count, how to calculate the total number of cells, and how to select specific cells to be counted as positive.^16^ During our quest for an intraoperative quantitative method to predict the risk of recurrence, we noted intratumoral heterogeneity in the proliferative potential of meningioma cells. Some previous studies failed to prove the predictive value of MIB‐1 LI,^32^, ^33^ which may be partially explained by its low quantitative capability and heterogeneity. Thus, iFC may be effective in precisely measuring the proliferative ability in each case, which we believe leads to a more accurate estimation of tumor behavior after surgery.

Our data demonstrate that the intratumoral heterogeneity was associated with some tumor biological characteristics, which raises two interesting discussion points. First, our results suggest the importance of the biopsy location for the accurate estimation of recurrence. Given the heterogeneity, specimens should be obtained from the residual mass or from regions close to the tumor attachment instead of being randomly obtained. Recently, the histological diagnosis of meningioma, especially for WHO Grade II and III meningiomas, has been a target of debate. The most recent update of the WHO classification recommends the evaluation of brain invasion.^34^ Examining the entire brain‐tumor interface is not always possible because most meningiomas are removed in a piecemeal fashion,^35^ which might lead to some inconsistency regarding the significance of brain invasion.^36^ Recently, the association of telomerase reverse transcriptase (TERT) promoter mutations with malignant progression in meningioma has been reported.^37^ Interestingly, spatial intratumoral heterogeneity of TERT promoter mutations was observed.^38^ Amid calls for the need to standardize the surgical sampling technique,^39^ evaluation of the proliferative potential of residual or affected tissues is obviously necessary for a more accurate estimation of recurrence, especially in situations where the possibility of non‐benign meningiomas is suspected. Second, the intratumoral heterogeneity appears to stem from the differences in the blood supply. Based on our results, meningiomas with pial feeders are more frequently associated with an elevated proliferative potential in the peripheral region of the tumor. The development of pial feeders may increase the vascular supply and result in higher proliferative potential.

One future perspective of the current study is that this quantitative method may be theoretically helpful for selecting patients who will benefit from upfront radiosurgery following subtotal resection. Postoperative radiation for benign meningiomas would not be conducted after gross total resection. Alternatively, adjuvant radiation is recommended after subtotal resection of WHO Grade II and III meningiomas.^40^ However, there has been no consensus on immediate radiosurgery after subtotal resection of WHO Grade I meningiomas. Recently, some rigorous attempts have been made to isolate the histologically benign meningiomas at significantly increased risk of recurrence. The revision regarding brain invasion in the latest criteria for WHO Grade II meningiomas^21^ is in line with this idea. Given that the recurrence rate of meningiomas with an MIB‐1 LI of 3% or higher is similar to that of WHO Grade II meningiomas,^4^, ^13^, ^41^ MIB‐1 LI is a candidate standard for the judgment on administering immediate radiosurgery, despite its inherent bias. Marciscano et al reported that WHO Grade I meningiomas with at least one atypical factor such as increased cellularity, sheeting, prominent nuclei, necrosis, and high nucleus‐to‐cytoplasm ratio carry a higher risk of recurrence when treated with Simpson Grade II‐IV resections compared to those with no atypical features.^3^ Along with these recent efforts, more quantitative and reproducible measurements of proliferative potential such as iFC would contribute to the refinement of treatment indications.

There are some limitations to the current study. Obviously, long‐term follow‐up is mandatory to check whether these intraoperative data pertain to the postoperative tumor growth behavior. Based on our experience, tumors with remarkable calcifications are not appropriate for iFC because of the debris generated during homogenization. We have little data on meningiomas treated with radiation or preoperative embolization. We believe that the conclusions of this study would not apply to these specimens given that they contain substantial necrosis. Nevertheless, information regarding the proliferative ability of each tumor will help individualize the surgical strategy. Accurate prediction of future recurrence enables refinement of postoperative treatment and follow‐up, which leads to improvement of the treatment outcome.

## 5 CONCLUSIONS

Our prospective study demonstrates two novel findings. First, the results of rapid iFC are correlated with postoperative MIB‐1 LI, preoperative AGR, and WHO grades. These data suggest that iFC is promising as an indicator that overcomes major drawbacks of MIB‐1 staining, such as low rapidity, and low quantitative ability, which may contribute to provide valuable information to surgeons for weighing the risks and benefits of aggressive surgery and achieve safe maximal resection. Second, intratumoral heterogeneity of the cellular proliferative potential exists in meningiomas and is related to their biological characteristics, underlining the importance of standardizing the sampling method to accurately estimate the risk of meningioma recurrence.

## CONFLICT OF INTEREST

This study was conducted under a collaborative research agreement between Saitama Medical University and the Nihon Kohden Corporation for the voluntary lease of the pipetting device used for tissue preparation.
